# Impact of HPV-16/18 AS04-adjuvanted vaccine on preventing subsequent infection and disease after excision treatment: post-hoc analysis from a randomized controlled trial

**DOI:** 10.1186/s12879-020-05560-z

**Published:** 2020-11-16

**Authors:** Shuang Zhao, Shangying Hu, Xiaoqian Xu, Xun Zhang, Qinjing Pan, Feng Chen, Fanghui Zhao

**Affiliations:** 1 Department of Epidemiology, National Cancer Center/ National Clinical Research Center for Cancer/Cancer Hospital, Chinese Academy of Medical Sciences and Peking Union Medical College, 17 South Panjiayuan Lane, PO Box 2258, Beijing, 100021 China; 2 Department of Cytology and Pathology, National Cancer Center/ National Clinical Research Center for Cancer/Cancer Hospital, Chinese Academy of Medical Sciences and Peking Union Medical College, Beijing, 100021 China

**Keywords:** Human papillomavirus, Cervical Cancer, Excision treatment, Clinical trial, Vaccine

## Abstract

**Background:**

It is widely acknowledged that HPV prophylactic vaccine could prevent new infections and their associated lesions among women who are predominantly HPV-naive at vaccination. Yet there still remains uncertainty about whether HPV vaccination could benefit to individuals who have undergone surgery for cervical disease.

**Methods:**

This post-hoc analysis intends to focus on intent-to-treat participants who underwent excision treatment at baseline and the follow-up period in a phase II/III, double-blind, randomized trial (ClinicalTrials.gov, number NCT00779766) conducted in Jiangsu province, China. We evaluate the impact of HPV vaccination on preventing women from subsequent infection and cervical lesions (LSIL+ and CIN2+) after excision treatment.

**Results:**

One hundred sixty-eight (vaccine, *n* = 87; placebo, *n* = 81) performed excisional treatment in this clinical trial. We observed a significant effect of vaccination on acquiring 14 high-risk HPV (HR-HPV) infection after treatment (vaccine efficacy: 27.0%; 95% CI 4.9, 44.0%). The vaccine efficacy against new infections after treatment for 14 HR-HPV infection was estimated as 32.0% (95%CI 1.8, 52.8%), and was 41.2% (95%CI -162.7, 86.8%) for HPV16/18 infection. The accumulative clearance rates of the vaccine group and placebo group were 88.9 and 81.6% for HPV16/18 infection (*P* = 0.345), 63.4, 48.7% for 14 HR-HPV infection (*P* = 0.062), respectively. No significant difference was observed on the persistent rate of HPV16/18, 14 HR-HPV infection and occurrence rate of LSIL+ between the two groups.

**Conclusions:**

No significant evidence from this study showed that HPV-16/18 AS04-adjuvanted vaccine could lead to viral faster clearance or have any effect on the rates of persistent infection among women who had excision treatment. However, the vaccine may still benefit post-treatment women with “primary prophylactic” effect. Further research is required in clarifying the effect of using the prophylactic HPV vaccine as therapeutic agents.

**Trial registration:**

**ClinicalTrials.gov identifier**: NCT00779766. **Date and status of trial registration:** October 24, 2008. Completed; Has Results.

## Background

Persistent high-risk human papillomavirus (HR-HPV) infection is necessary for the progression of cervical cancer [1, 2]. The development of prophylactic HPV vaccines has led a momentous positive impact on cervical cancer prevention and control. High efficacy of HPV vaccine has been demonstrated against cervical intraepithelial neoplasia (CIN) grade II/III among women who are HPV-naive at vaccination in multiple studies [3, 4]. However, the impact of prophylactic vaccine on women who have been previously treated for CIN has not been fully understood.

Women after treatment for CIN remain at a substantially increased risk of subsequent cervical cancer [5–8]. Both randomized and non-randomized studies have indicated a potentially positive effect of HPV vaccination on women who had been treated for precancerous lesions or cancers [9–12]. A post-hoc analysis of a large randomized phase III trials showed a significant reduction on relapse of any subsequent high grade cervical disease (64.9%) among post-treatment women who previously received the quadrivalent HPV vaccine [9]. A prospective study, evaluating the clinical effectiveness of HPV vaccine in reducing CIN2+ recurrent disease among women who underwent cervical conization for cervical HSIL and FIGO stage Ia1 cervical cancer, suggested that quadrivalent HPV vaccination could reduce the risk of subsequent HPV related high-grade CIN after cervical surgery by 81.2% [12].

However, few studies evaluate whether the observed effects arise from: (1) “therapeutic effect” of the prophylactic vaccination in leading to faster clearance of the residual infection; or (2) “primary prophylactic” effect of the vaccination on the newly developed lesions caused by new HPV infections after treatment; or (3) “secondary prophylactic” effect of the vaccination in reducing the ability of the residual virus to infect new cells. In this study, we explore the role of a bivalent vaccine in preventing secondary lesions and provide more scientific evidence for the impact of the vaccine on the women who are treated for cervical disease.

## Methods

### Eligible participants

Women included in the present evaluation were the participants who underwent excision treatment at baseline and the follow-up period in a phase II/III, double-blind, randomized trial conducted in Jiangsu province (Binhai, Jintan, Lianshui and Xuzhou CDCs), China. In this trial, women aged 18–25 years were randomized (1:1) to receive HPV-16/18 AS04-adjuvanted vaccine(*n* = 3026) or aluminum hydroxide (*n* = 3025) as a placebo at months 0, 1 and 6. Enrolment in this trial started in October 2008 and follow-up lasted for 72 months (14 visits). The trial was conducted according to The Code of Ethics of the World Medical Association (Declaration of Helsinki) and the International Conference on Harmonisation Good Clinical Practice guidelines. The study protocol and informed consent were approved by the ethics committees of the Center for Disease Control and Prevention (CDC) Jiangsu Province and the Cancer Foundation of China. The trial was registered at the ClinicalTrials.gov (number NCT00779766) and adhered to CONSORT guidelines. Before the study-specific procedures, written informed consent was obtained from each participant. The further details of the trial have been described in previous published papers [13–15]. Briefly, women after excision treatment were followed up by collecting cervical exfoliated cell samples in gynecologic physical examination at each study visit. The cervical samples were evaluated by HPV DNA PCR testing and cytology. For all cytology diagnoses of Atypical Squamous Cells of Undetermined Significance (ASCUS), the central laboratory had additionally performed HC2 High-Risk HPV DNA Test™ (Qiagen Inc., Gaithersburg, MD) on residual PreservCyt® specimen. The subjects were referred to colposcopy if they had cytology ASCUS with HPV positive results (by HC2 HPV DNA test), or cytology low-grade squamous cell intraepithelial lesion or worse (LSIL+) independent of HPV DNA results.

**Fig. 1:**
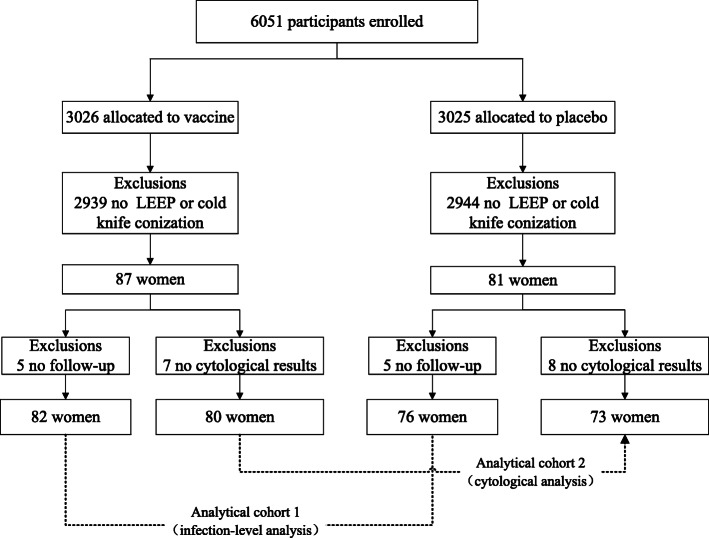
Participant disposition. Abbreviations: LEEP: loop electrosurgical excision procedure

In this post-hoc analysis, we intend to explore the impact of HPV vaccination on women after excision treatment by focusing on intent-to-treat participants who received at least one dose of vaccine or placebo. Three level of analysis would be conducted in focusing on HPV infection (Fig. 1: analytical cohort 1), LSIL analysis (Fig. 1: analytical cohort 2) and recurrence of precancerous lesions, respectively. In the HPV infection-level analysis, each infection instead of a woman was considered as the unit of analysis to estimate the incidence rate of HPV16/18 (vaccine-specific types), HPV31/33/45(cross-protective types) [16], 14 HR-HPV (any of the 14 high-risk HPV types) and 11 LR-HPV (any of the 11 low-risk HPV types) infection. Persistent infection was defined as at least two positive HPV DNA PCR assays for the same viral genotype with no negative DNA sample between the two positive DNA samples, over an interval of 6 months or longer. Newly acquired infection (new infection) was defined as the positive detection by PCR of an episode of infection by HPV type(s) in a subject who was negative at excision treatment visit for the considered HPV type(s). Given the high efficacy of the HPV-16/18 vaccine against new infections, we also conducted analysis restricted to newly detected infection after treatment.

### Cytology and histopathology

Cervical exfoliated cell samples for HPV DNA testing and cytological evaluation were collected at each study visit. Cytological evaluation was performed at the Cancer Institute of the Chinese Academy of Medical Sciences (CICAMS). And the results were interpreted according to the Bethesda 2001 classification system [17]. Participants with abnormal results were managed in accordance with the pre-specified algorithms, which had been described previously [13–15]. Biopsy and excisional treatment specimens were analyzed by a panel consisted of three expert gynecological pathologists. The data for women with CIN diagnoses were reviewed by an independent endpoint committee who had been blinded to make final case assignments.

### HPV DNA testing

The automatic analyzer SPF10 PCR-DEIA-LiPA25 version 1 (manufactured by Labo Biomedial Product, Rijswijk, the Netherlands based on licensed Innogenetics technology) detected HPV DNA of cervical, biopsy samples and tissue specimens including 14 high-risk HPV types (16, 18, 31, 33, 35, 39, 45, 51, 52, 56, 58, 59, 66 and 68) and 11 low-risk HPV types (6, 11, 34, 40, 42, 43, 44, 53, 54, 70 and 74).

### Statistical analysis

Comparisons of enrollment characteristic, accumulative clearance rate and persistent infection rate between the two groups were conducted by Pearson χ^2^ or Fisher’s exact tests, as appropriate. Vaccine efficacy (VE) was calculated as 1-(incidence rate in vaccinated/incidence rate in unvaccinated) and the corresponding 95% confidence intervals (95% CIs) around vaccine efficacy were calculated by exact conditional procedure based on the observed case split between vaccine and placebo recipients, adjusted for the person time in each arm. In the squamous intraepithelial lesion (SIL) analysis, we analyzed the occurrence rate of LSIL+ and HSIL+ stratified by specific-type after excisional procedure. Results of vaccine efficacy were considered as statistically significant if the estimates and corresponding 95% CIs were above zero. Kaplan-Meier method with log-rank test was applied to compare the difference in occurrence of LSIL+ between the two groups. All statistical tests were two-sided and only *p*-values < 0.05 were considered statistically significant. The statistical analyses were performed using WinPEPI version 4.0 [18] or SPSS 23.0 (SPSS, Inc., Chicago, Illinois).

## Results

### Enrollment and follow-up characteristic of women after cervical surgery

168 women (vaccine, *n* = 87; placebo, *n* = 81) received excisional treatment for their first cervical lesion identified at this clinical trial. One woman in each group underwent loop electrosurgical excision procedure (LEEP) at the last visit (visit 14), and thus 166 women had been followed up and were categorized based on cytological results and HPV status at enrollment. Comparison of analytical cohort stratified by study arm revealed the balance regarding to cytology results, and HPV status (HPV type) at enrollment (Table 1).

**Table 1:**
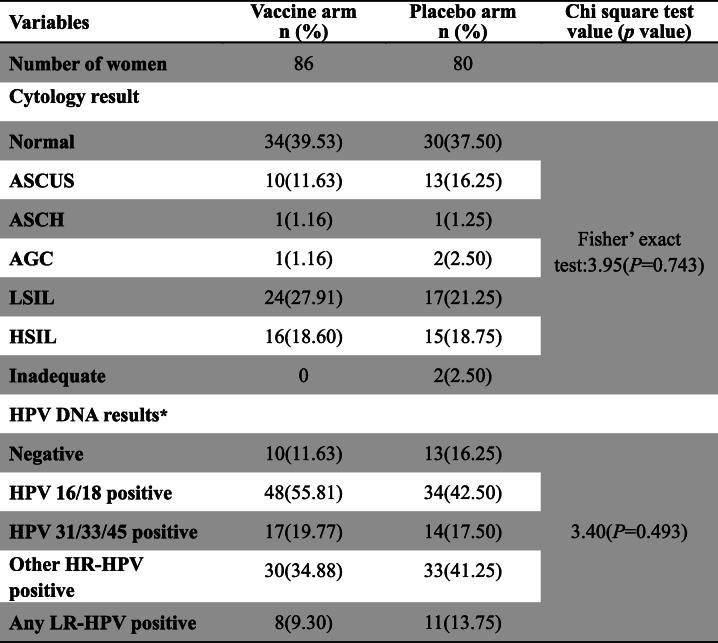
Enrollment characteristic of women who had undergone cervical surgery after randomization to HPV-16/18 AS04-adjuvanted vaccine or placebo. * The total percentages of each HPV type is not necessarily equal to 100% because a result may be counted more than once in cases where the participants contained multiple HPV type. ASCUS: atypical squamous cells of undetermined significance; ASCH: atypical squamous cell, but cannot exclude high-grade squamous intraepithelial lesion; AGC: atypical glandular cells; LSIL: low-grade squamous intraepithelial lesion; HSIL: high-grade squamous intraepithelial lesion; Inadequate: unsatisfactory cytological results; HPV: human papillomavirus; HR-HPV: high-risk human papillomavirus; LR-HPV: low-risk human papillomavirus

Women who underwent cervical treatment surgery were followed for a median of 50.0 months after treatment (vaccine arm: 49.5, IQR: 32.0–64.0; placebo arm: 50.0, IQR: 30.3–63.5), corresponding to a median of 9 study visits (vaccine arm: 9, IQR: 5–12; placebo arm: 8, IQR: 5–11). The median duration between enrollment and treatment was 17 months for those in the vaccine arm (IQR:4.0–30.0 months) and 17 months for women in the placebo arm (IQR:6.0–34.8 months).

### Impact of vaccination on rate of HPV infections and abnormal cytological results after excision treatment

In the infection-level analysis, 10 women (vaccine, *n* = 5; placebo, n = 5) were excluded because of no follow-up after excision treatment (Fig. 1). Finally, 158 women (vaccine, *n* = 82; placebo, *n* = 76) were included in the analysis. There was no significant difference in the distribution of baseline HPV infection between the two groups (Fisher’ exact test: 3.64, *P* = 0.458). The woman was included in this analysis from the day when the women received an excisional procedure (LEEP or cone) for a first cervical lesion to the day of their last follow-up visit, considering the fact that women could be infected by HPV or had abnormal cytological results for several times after excisional treatment. Among all the infections that treated women had, 71.06% were HR-HPV infection and of these 52.49% were the result of new infections. We observed a significant effect of vaccination on acquiring 14 HR-HPV infection (VE 27.0%; 95%CI 4.9, 44.0%), and a nonsignificant but positive vaccine efficacy estimate of 28.1% (95% CI -62.9,68.3%) for HPV16/18 infection after excision treatment (Table 2). Vaccine efficacy against new infections after treatment for 14 HR-HPV infection was estimated to be 32.0% (95%CI 1.8,52.8%), and was 41.2% (95%CI -162.7,86.8%) for HPV-16/18 infection, which was consistently positive in this restricted analysis but didn’t reach statistical significance.

**Table 2:**
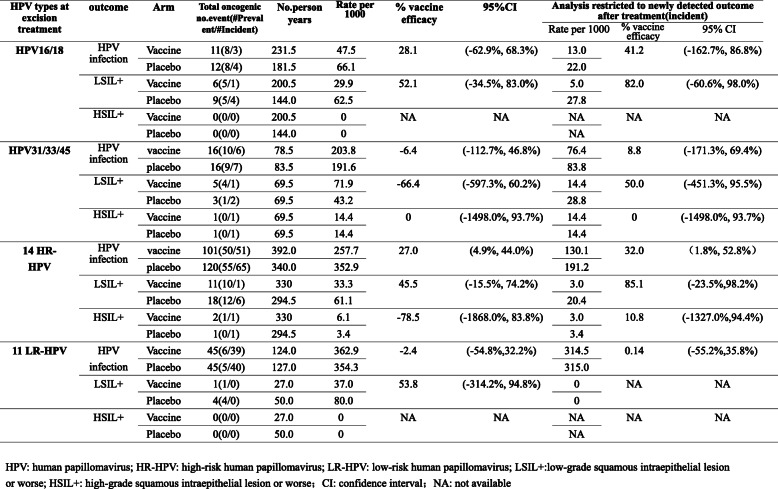
Impact of HPV-16/18 vaccination on recurrence of HPV infections and cervical lesions after excision treatment. HPV: human papillomavirus; HR-HPV: high-risk human papillomavirus; LR-HPV: low-risk human papillomavirus; LSIL+:low-grade squamous intraepithelial lesion or worse; HSIL+: high-grade squamous intraepithelial lesion or worse; CI: confidence interval; NA: not available

**Fig. 2:**
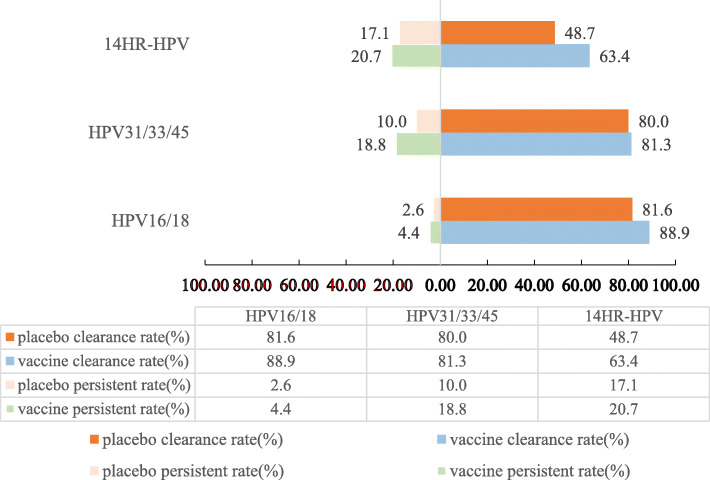
Impact of HPV-16/18 AS04-adjuvanted vaccine on accumulative clearance rate and persistent infection rate among the women after excision treatment

Then, we evaluated whether the viral clearance rates or HPV persistent infection rates differed by vaccination status. Results were shown in Fig. 2. No evidence from this study showed that vaccination led to viral faster clearance or had any effect on the persistent infection rate. The accumulative clearance rates of the vaccine group and placebo group were 88.9, 81.6% for HPV16/18 infection(*P* = 0.345), 81.3, 80.0% for HPV31/33/45 infection(*P*>0.999), 63.4, 48.7% for 14 HR-HPV infection(*P* = 0.062), respectively. No significant difference on the persistent infection rate of HPV16/18(4.4% vs 2.6%,*P*>0.999), HPV31/33/45(18.8% vs 10.0%,*P* = 0.637),14 HR-HPV (20.7% vs 17.1%,*P* = 0.561) was identified between the two groups.

When LSIL+ was examined as the outcome, nonsignificant but positive vaccine efficacy was estimated at 45.5% (95%CI -15.5, 74.2%) for 14 HR-HPV infection, and 85.1% (95% CI -23.5, 98.2%) for the newly detected outcome after treatment, respectively. Similar patterns were observed for HPV-16/18 infection as well.

### Impact of vaccination on occurrence of LSIL+ and subsequent histopathologically confirmed disease

**Fig. 3:**
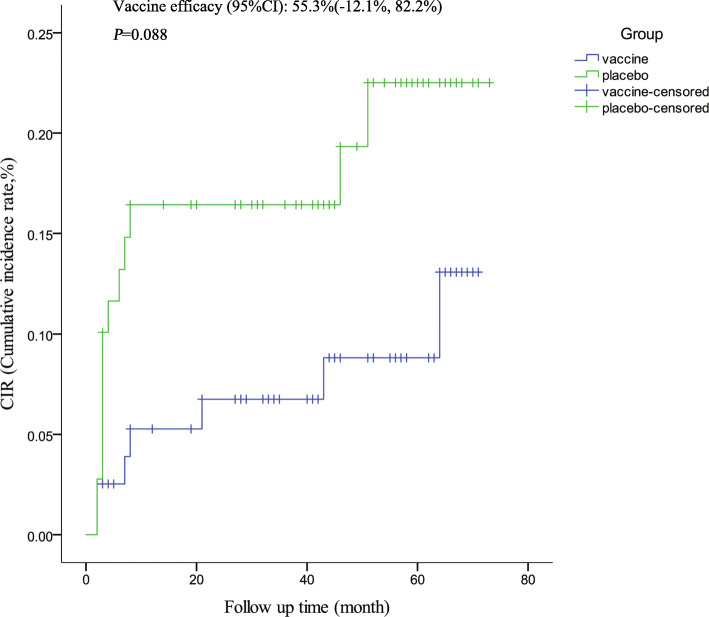
Impact of HPV-16/18 AS04-adjuvanted vaccine on occurrence of low-grade squamous intraepithelial lesion or worse (LSIL+)

15 women (vaccine, *n* = 7; placebo, *n* = 8) were excluded because of no cytological results (Fig. 1). Finally, 153 women were included in the final analysis (vaccine, *n* = 80; placebo, *n* = 73). The distribution of cytological results at baseline was similar between two groups (Fisher’ exact test: 8.80, *P* = 0.051). In this analysis, the follow-up time was defined as the duration from the day of excisional procedure to the day of ascertainment of the subsequent disease end point (LSIL+ incidence), while for women without a subsequent disease end point, until the day of their last follow-up visit. The median follow-up time was 46.0 months among women who were treated. The median follow-up time for vaccine group and placebo group were 48.5 months, 44.0 months, respectively. The vaccine efficacy for the occurrence of LSIL+ was 55.3% (95%CI -12.1, 82.2%), irrespective of HPV DNA types. There was no significant difference between two groups (*P* = 0.088, Fig. 3).

**Fig. 4:**
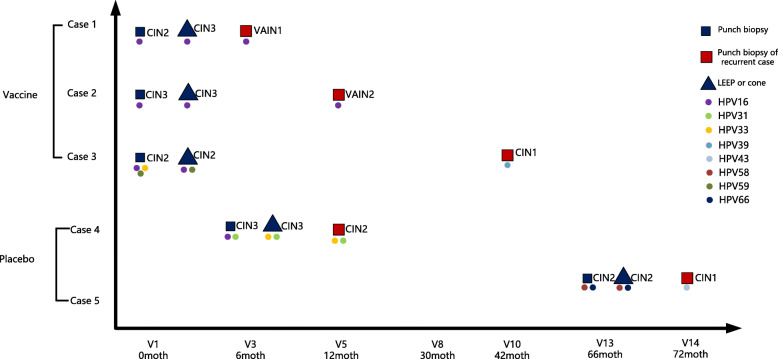
Biopsy type, histopathological diagnosis and HPV DNA result in lesion for women who had undergone surgical therapy for cervical disease. Case 1: the subject was HPV-16, 52 DNA positive, with ASCUS predicted by cytology at gynecological examination at visit 1. Case 2: occurred in a woman who, at baseline (visit1), was HPV-16 DNA positive, with HSIL predicted by cytology. Case 3: the woman was HPV-16, 59 DNA positive, with LSIL predicted by cytology at visit 1.And the subject was high-risk HPV DNA negative at month 18. HPV-39 was detected at month 30. Case 4: the subject was HPV-16, 31, 33 DNA positive, with HSIL predicted by cytology at gynecological examination at month 6. Case 5: the subject was HPV-58 DNA positive, with LSIL predicted by cytology at gynecological examination at month 66. Abbreviations: CIN: cervical intraepithelial neoplasia; VAIN: vaginal intraepithelial neoplasia; LEEP: loop electrosurgical excision procedure

In the vaccine group, one woman had been detected with vaginal intraepithelial neoplasia grade 2 (VAIN2), one with VAIN1, and one with CIN1 post-surgery. Of the two women in the placebo group, one developed CIN2 and one had CIN1 after treatment. The women (Case 4: Fig. 4) who developed CIN2 in the placebo group was HPV16, 31, 33 DNA positive, with HSIL predicted by cytology at gynecological examination at visit3 (6 month). She underwent cold knife conization treatment at visit 3(6 months) and CIN3 was diagnosed by histology; the margins of the excisional material were disease-free. After 6 months she had cytological ASCH (atypical squamous cell, but cannot exclude high-grade squamous intraepithelial lesion) results and was referred to colposcopy. CIN2 was diagnosed on punch biopsy and HPV genotyping on cervical tissue sample revealed HPV31, 33 positive. The one VAIN2 case in the vaccine group (Case 2: Fig. 4) occurred in a woman who was HPV16 DNA positive, with a cytology HSIL result at baseline (visit1). She underwent LEEP treatment at baseline (visit1) and CIN3 was diagnosed by histology; the margins of the excisional material were disease-free. At 12 months, she had ASCUS on cytology and was referred to colposcopy. VAIN2 was diagnosed on punch biopsy and HPV genotyping on cervical tissue sample revealed HPV16 positive.

## Discussion

This is the first analysis in China to evaluate the efficacy of HPV 16/18 AS04-adjuvanted vaccination on the relapse of cervical precancerous lesions or cancers among women who underwent excisional procedures for cervical lesions after vaccination. In this study, vaccination failed to lead to viral faster clearance or have any effect on persistent HPV infection. However, the available evidence suggested that the vaccine may still benefit women after excisional treatment for cervical disease by “primary prophylactic” effect, which protects women from new infection after treatment.

Former studies have indicated that the prophylactic HPV vaccines have the possibility in benefiting post-treatment women. The post-hoc analysis of the PApilloma TRIal against Cancer In young Adults (PATRICIA) showed that efficacy of the HPV 16/18 AS04-adjuvanted vaccine post-surgery (60 days or more) for the original lesion was 88.2% against CIN2+ and 42.6% against CIN1+ [19]. Furthermore, the analysis from a large randomized clinical trial in Costa Rica indicated significant vaccine efficacy against the development of new infection associated with HPV31/33/45 and any of the 12 HR-HPV types. Vaccine efficacy against HPV16/18 new infection was positive but didn’t reach statistical significance, which was consistent with our analysis [20]. Additionally, we found that HPV 16/18 AS04-adjuvanted vaccination could protect against any of the 14 HR-HPV infection and prevent its new infection after treatment. From our analysis, no significant difference for the accumulative clearance and persistent rate of HPV infection between two groups was identified. Women who underwent surgery for the first cervical lesions after receiving the vaccine may benefit from the vaccination, which could be attributed to the protect effects against new infections after treatment. Besides, a non-randomized observational study also demonstrated that quadrivalent vaccination after treatment of CIN2–3 significantly reduced the risk of recurrence in patients related to HPV16/18, which suggested that a benefit effect in offering HPV vaccination to women post CIN treatment [10].

The women who were treated due to HPV-related disease were at high risk for developing subsequent HPV related disease. The persistent or recurrent rate of CIN2+ after excisional treatment was reported as 4% ~ 18% [21]. A meta-analysis revealed that the risk of residual or recurrent CIN2+ was significantly increased among women with positive excision margins compared to those with negative margins [22]. The recurrence of histologically proven CIN2+ after the treatment for the first high-grade cervical lesion was influenced by many factors including initial diagnosis, age, treatment type and the infection status of original excisional margins. Another systematic review, reporting the data about newly detected HPV genotypes post-treatment for precancerous cervical lesions, showed the incident detection rate were estimated up to 24% for HR-HPV types at 3-11 months after treatment and up to 21% at 12–36 months [23]. These infections were most potentially newly acquired or reactivated from latent infections (i.e. detection of infection formerly present at levels below the cut-off point of the HPV assay). Therefore, potentially, neutralizing antibodies induced by vaccination could bind to virions newly acquired or virions produced by infected cells, reducing spread of an existing infection by restraining the ability of the residual virus to infect new cells and thereby play the protective role among women after excision treatment. Our study corroborated that post-treatment women may benefit from the protection against new infection after treatment. Further studies are needed to confirm the hypothesis whether the vaccine could prevent reactivation of latent infection and reduce spread of an existing infection by restraining the ability of the virus to infect new cells.

The major advantage of our study is that we have detailed information about the margin status of excisional material for each woman treated for the first cervical lesion who subsequently developed lesions after surgery. This made it possible to confirm whether subsequent cervical lesions were associated with HPV genotype infection found in the original lesion. However, there are some limitations in our analysis. The subgroup of women who underwent excision treatment was not a randomized group, so we had limited power to evaluate post-treatment vaccine efficacy. Furthermore, because no any sexual behavior data was collected, we were not able to evaluate the sexual behavior difference between two groups or estimate the effect of vaccination by adjusting the factor. However, a good balance regarding HPV status at enrollment had been achieved between the two arms, which indicated that there may be no significant difference in sexual behavior between the two groups. And we used cytological results as an outcome in the analysis due to not all women after treatment underwent colposcopy, which may have potential bias. Based on previous study [24], cytology showed the higher specificity at the cost of lower sensitivity compared to colposcopy, so women without distinct cytological performance would be missed. Additionally, due to the small number of women who received surgery in clinical trials and the fact that loss of follow-up further reduced sample size included in the analysis, we had limited statistical power. There remains a need for more studies with a larger sample size to further confirm the post-treatment vaccine efficacy against new infections and resultant lesions.

## Conclusions

Significant vaccine efficacy against new infections after treatment for 14 HR-HPV infection was observed. The nonsignificant effect against new infections associated with HPV-16/18 and cervical lesions (LSIL+ and CIN2+) maybe caused by the limited power with smaller number of women. However, the vaccine may still benefit post-treatment women with the “primary prophylactic” effect. Further research is required in clarifying the effect of using the prophylactic HPV vaccine as therapeutic agents.

## Data Availability

Anonymized individual participant data and study documents can be requested for further research from www.clinicalstudydatarequest.com.

## Abbreviations

HPV: Human Papillomavirus
HR-HPV: High Risk Human Papillomavirus
LR-HPV: Low Risk Human Papillomavirus
LEEP: Loop Electrosurgical Excision Procedure
CIN: Cervical Intraepithelial Neoplasia
IQR: Interquartile Range
CICAMS: Cancer Institute of the Chinese Academy of Medical Sciences
VE: Vaccine Efficacy
VAIN: Vaginal Intraepithelial Neoplasia
ASCUS: Atypical Squamous Cells of Undetermined Significance
ASCH: Atypical Squamous Cell, but cannot exclude high-grade squamous intraepithelial lesion
AGC: Atypical Glandular Cells
LSIL+: Low-grade Squamous Intraepithelial Lesion or worse
HSIL+: High-grade Squamous Intraepithelial Lesion or worse
CI: Confidence Interval
